# Temporal evolution of quantitative EEG within 3 days of birth in early preterm infants

**DOI:** 10.1038/s41598-019-41227-9

**Published:** 2019-03-19

**Authors:** John M. O’Toole, Elena Pavlidis, Irina Korotchikova, Geraldine B. Boylan, Nathan J. Stevenson

**Affiliations:** 1 Neonatal Brain Research Group, Irish Centre for Fetal and Neonatal Translational Research (INFANT), University College Cork, Cork, Ireland; 2 Department of Paediatrics and Child Health, University College Cork, Cork, Ireland; 3 BABA Center, Department of Children’s Clinical Neurophysiology, Children’s Hospital, HUS Medical Imaging Center, Helsinki University Central Hospital and University of Helsinki, Helsinki, Finland

## Abstract

For the premature newborn, little is known about changes in brain activity during transition to extra-uterine life. We aim to quantify these changes in relation to the longer-term maturation of the developing brain. We analysed EEG for up to 72 hours after birth from 28 infants born <32 weeks of gestation. These infants had favourable neurodevelopment at 2 years of age and were without significant neurological compromise at time of EEG monitoring. Quantitative EEG was generated using features representing EEG power, discontinuity, spectral distribution, and inter-hemispheric connectivity. We found rapid changes in cortical activity over the 3 days distinct from slower changes associated with gestational age: for many features, evolution over 1 day after birth is equivalent to approximately 1 to 2.5 weeks of maturation. Considerable changes in the EEG immediately after birth implies that postnatal adaption significantly influences cerebral activity for early preterm infants. Postnatal age, in addition to gestational age, should be considered when analysing preterm EEG within the first few days after birth.

## Introduction

For the newborn preterm infant, many physiological changes occur during the transition at birth from intra- to extra-uterine life. For example, haemodynamic instability during this period can lead to brain injury, with incidence rates as high as 1 in 3 for infants born <32 weeks of gestation^1^. Monitoring brain function can help the clinician identify infants at risk of injury and inform treatment strategies. Quantitative evaluation of neurological function can maximise the utility of existing monitoring technologies in the neonatal intensive care unit (NICU). Renewed interest in this area highlights the potential of a quantitative approach to improve clinical outcomes for these infants^2–7^.

The most commonly used method of brain monitoring in the NICU is the electroencephalogram (EEG). Between 24 and 32 weeks of gestation EEG activity varies considerably with gestational age (GA)^8–17^ reflecting the rapid increase in cerebral cortical volume^18^ and the process of neuronal organisation that begins at approximately five months of gestation^19^. One of the milestones of this process is the establishment and differentiation of the subplate neurons that provide the site of synaptic contact for thalamocortical and corticortical afferents before the cortical plate is formed and serve as the functional link between “waiting” afferents and cortical connections^19,20^. Quantitative analysis of the EEG, which involves extracting multiple statistical and signal-processing features from the EEG, provides objective and consistent analysis of this complex EEG. Quantitative EEG (qEEG) is also used to develop fully automated methods of EEG analysis; qEEG measures are used in methods to estimate maturation^7,17^ and classify sleep states^6,21^, for example. Such systems can integrate cotside and provide continuous information on cerebral function to the non-expert.

Although preterm EEG is well defined in relation to GA, little is known about how it evolves in the immediate period after birth. Many environmental factors, including the birth process, may have a significant impact on early postnatal adaptation^22–24^. Existing qEEG studies indicate that some features change during this period, and this change may be specific to GA^25–27^. The small number of non-specific qEEG features used in these studies, without features of spatial and temporal organisation for example, are not comprehensive enough to capture the complexity of the preterm EEG. In addition, quantification of the change in qEEG over time is lacking.

Quantifying the evolution of preterm EEG after birth in relation to intra-uterine maturation is important for both the visual and quantitative analysis of the EEG, particularly as dysmaturity of the EEG is a prominent diagnostic biomarker^5,28,29^. Furthermore, the EEG during postnatal adaption may be an important biomarker of neurological function that is complementary to longitudinal EEG monitoring. We hypothesize that the biological and environmental factors associated with preterm birth will alter the typical intra-uterine maturation of the EEG to produce rapid changes in the EEG. To test this hypothesis, we aim to quantify the temporal evolution of qEEG over the first 3 days after birth for preterm infants <32 weeks of gestation and quantify the role GA may play in this evolution.

## Results

**Figure 1:**
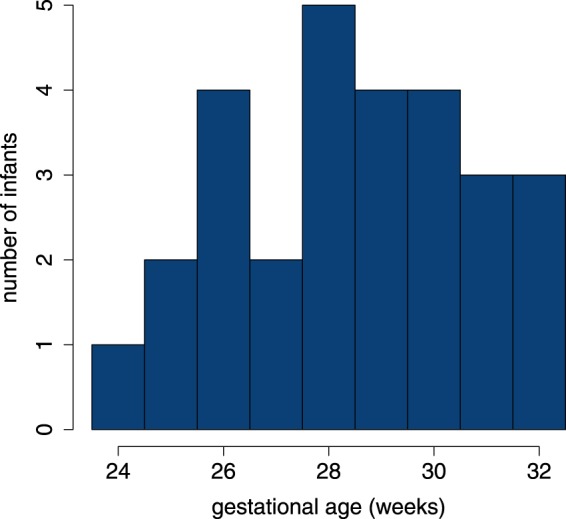
Distribution of gestational age.

Twenty-eight infants met the inclusion criteria and were included in this study. Table 1 summarises the clinical characteristics for this cohort. Figure 1 shows the distribution of GA with median age of 28.5 weeks, range 24.0 to 31.9 weeks.

**Table 1:** Patient characteristics presented as either median (inter-quartile range) or sample size (%).

characteristic	*n* = 28
female	20 (71.4%)
gestational age (weeks)	28.5 (26.6, 30.1)
Apgar score at 1 m	8 (6, 8.25)
Apgar score at 5 m	9 (8, 9)
birth weight (g)	990 (785, 1250)
pH	7.22 (7.15, 7.28)
cesarean delivery	13 (46%)
CRIB^†^	8 (7, 10)
medication^‡^	
morphine	0 (0%)
caffeine	27 (96%)
surfactant	18 (64%)
fentanyl and suxamethonium	6 (21%)
Bayleys III	
cognitive	95 (95, 103.8)
motor	100 (97, 106)
language	103 (97, 111.3)

^†^Clinical risk index for babies (CRIB) II generated for *n* = 26. ^‡^Administered <72 hours after birth.

Median postnatal age (PNA) at the start of EEG recording was 7.8 h (inter-quartile range, IQR: 5.0 to 17.1 h; range: 3.6 to 61.9 h). Median duration of continuous EEG recording was 40.5 h (IQR: 24.2 to 48.0 h; range 4.4 to 71.1 h). The artefact-detection algorithm (O’Toole *et al*.^30^) rejected a median of 0.1% (IQR: 0 to 1.1%; range: 0 to 28.7%) of the EEG in each epoch. Median number of channels rejected by this algorithm, per epoch, was 0 (IQR: 0 to 1; range: 0 to 8). If 8 channels were identified as artefact, the epoch was removed from further analysis. As there was only 2 epochs at the 72 h time-point, we removed this time point while keeping the remaining 6, 12, 24, 36, 48, and 60 h time points. After EEG epoch selection, the median number of epochs per infant was 3.5 (IQR: 3 to 5; range: 1 to 6).

### Fitting mixed-effects models

Fixed-effects estimates, including 95% confidence intervals, for all 34 features are presented in Tables 2 and 3. Most features are dependent on GA: 27/34 (79.4%) of the models include GA as a fixed effect. Over one-half of the features are dependent on PNA, with 19/34 (55.9%) models including PNA as a fixed effect. For this subset of 19 PNA-dependent features, our modelling approach allows for 3 types of temporal evolution.

**Table 2:** Estimates (95% confidence intervals) of fixed-effect coefficients for EEG features in the power and discontinuity groups. Coefficients of the mixed-model include intercept, gestational age (GA, age at birth in weeks), postnatal age (PNA, time after birth in days), and the interaction term between PNA and GA (PNA × GA).

Feature Type				Coefficients			
group	Feature	FB	C_th_	Intercept	PNA (days)	GA (weeks)	PNA × GA
power	SP (*μV*^2^)	*δ*		−177 (−1519, 1142)	1222 (600, 1821)	18 (−30, 65)	−38 (−60, −16)
		*θ*		30 (−38, 94)	33 (9, 59)	−0.36 (−2.62, 2.01)	−10 (−2, 0)
		*α*		4.8 (3.7, 5.8)	1.1 (0.5, 1.7)		
		*β*		1.6 (1.1, 2.1)	0.59 (0.24, 0.99)		
	rEEG (*μV*)		50th	24 (20, 27)	5.2 (3.4, 7.1)		
			5th	−7.6 (−16.3, 1.3)	1.2 (0.1, 2.1)	0.58 (0.27, 0.9)	
			95th	93 (−154, 334)	175 (54, 298)	0.98 (−7.55, 9.79)	−5.9 (−10.2, −1.4)
discontinuity	r-AS			1.4 (1.2, 1.7)	−0.037 (−0.058, −0.016)	−0.027 (−0.036, −0.018)	
	skew	*δ*		0.60 (−0.36, 1.55)	0.72 (0.13, 1.32)	−0.73e-3 (−35e-3, 33e-3)	−0.026 (−0.047, −0.005)
		*θ*		0.60 (0.47, 0.74)	−0.015 (−0.026, −0.003)	−0.014 (−0.019, −0.01)	
		*α*		0.15 (0.13, 0.18)		−4.2e-3 (−5.0e-3, −3.4e-3)	
		*β*		0.89 (0.67, 1.09)		−0.023 (−0.031, −0.016)	
	kurtosis	*δ*		26 (19, 32)		−0.62 (−0.84, −0.4)	
		*θ*		78 (61, 95)	−2.5 (−4, −1.1)	−1.9 (−2.5, −1.3)	
		*α*		86 (65, 106)		−2.3 (−3, −1.6)	
		*β*		83 (61, 106)		−2.2 (−3.1, −1.5)	
	IBI (s)		95th	35 (24, 47)	−0.99 (−1.84, −0.11)	−0.81 (−1.23, −0.44)	
			50th	6.2 (3.9, 8.5)		−0.12 (−0.2, −0.04)	
	burst% (%)			3.3 (−34.9, 39.2)	5.8 (2.7, 8.7)	2.3 (1, 3.6)	
	burst#			516 (368, 659)	−27 (−44, −11)	−10 (−15, −5)	

Frequency bands (FB): 0.5–3 Hz (*δ*), 3–8 Hz (*θ*), 8–15 Hz (*α*), and 15–30 Hz (*β*); C_th_: centile. rEEG features (median, lower- and upper-margins) are described by corresponding centiles (50th, 5th, and 95th), as is median and maximum inter-burst intervals (50th and 95th centile). SP: spectral power; rEEG: range EEG; r-AS: rEEG asymmetry; IBI: inter-burst interval; burst%: burst ratio; burst#: number of bursts.

**Figure 2:**
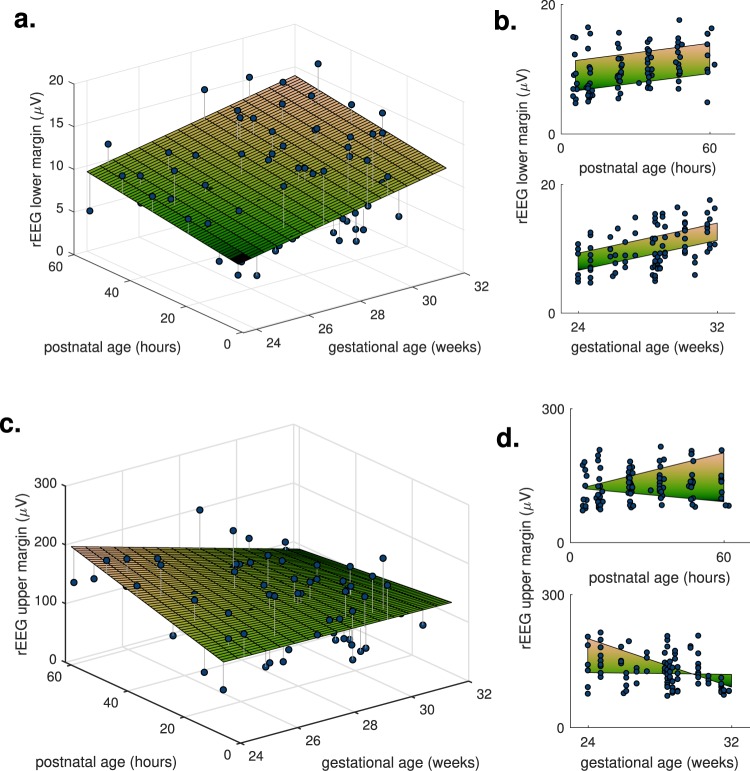
Two features of the rEEG, lower and upper margins, as dots with estimated fixed effects (2D planes). Features are dependent on both gestational age and postnatal age as the 2D plots in (**a**,**c**) illustrate this dependency; plots (**b**,**d**) show the marginal distribution, the side-view from either the x- or y-axis of the 2D plots in (**a**,**c**). The rEEG upper margin includes the postnatal age–by–gestational age fixed effect, resulting in a nonlinear 2D plane.

First, the model also includes GA, resulting in 2D trajectories over PNA and GA, such as the rEEG lower-margin feature in Fig. 2(a,b). Almost one-half (9/19) of the features are in this group. Second, the model includes the PNA–GA interaction term, resulting in diverging trends for different GAs, such as the upper-margin rEEG feature in Fig. 2(c,d). A smaller proportion (6/19) of the features fit this model type. And third, the model is dependent on PNA but independent of GA. A low number (4/19) of features meet this type, in keeping with the total percentage (20.5%) of features that are independent of GA.

**Figure 3:**
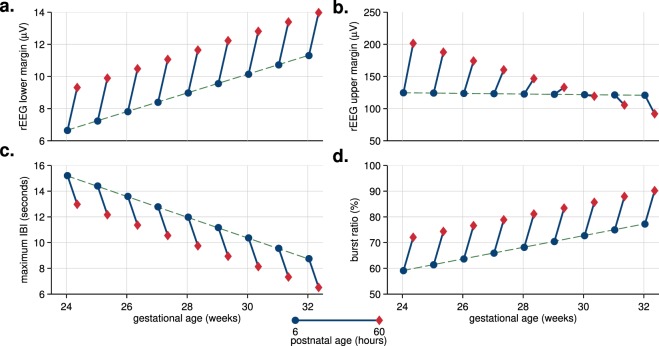
Visualising the temporal trajectories in context of intra-uterine maturation: plots of postnatal-age fixed-effect (as dots-to-diamonds lines) at selected gestational ages. Four features, 2 from the range-EEG (rEEG) in (**a**,**b**) and 2 features of the burst annotation (**c**,**d**); maximum IBI (inter-burst interval) refers to the 95th centile of the IBI. All features are dependent on time and gestational age; rEEG upper margin in (**b**) is also dependent on time-by-gestational interaction.

To aid comprehension of the different model types, Fig. 3 presents an alternative visualisation of the temporal trajectories. These plots show the fixed-effect estimates of PNA plotted in context of increasing GA. For the first group (models with both PNA and GA terms), the change (trend) in PNA is the same but the starting point for this change is dependent on GA. Figure 3(a,c,d) show examples of these types of trajectories. For the second group (models including the PNA–GA interaction term), again the starting point of the PNA trend changes with GA, but also the direction of trend itself changes with increasing GA. Figure 3(b) shows an example of this trajectory type. Plots for all 6 models that include the interaction term are in Supplementary Fig. S1.

**Figure 4:**
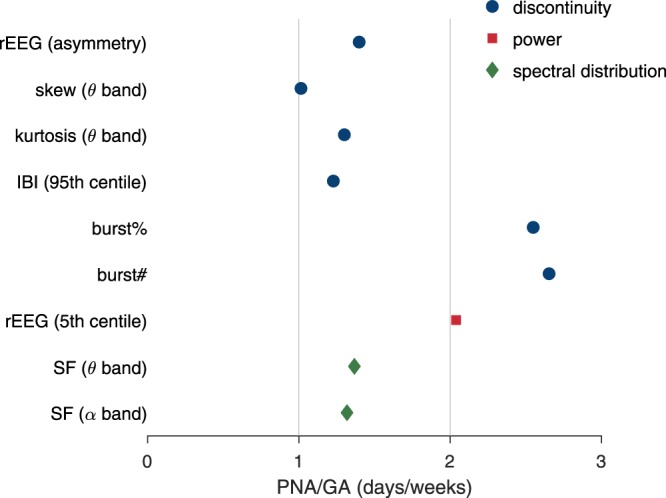
Ratio of estimated fixed-effects of postnatal age (PNA) to gestational age (GA). SF: spectral flatness; rEEG: range EEG; IBI: inter-burst interval; burst%: burst ratio; burst#: number of bursts. Frequency bands defined as 3–8 Hz for *θ* and 8–15 Hz for *α* bands.

Figure 3 shows that the relative change in hours after birth (PNA) is comparable to weeks of intra-uterine maturation (GA). To quantify this relative change, we assess the ratio between postnatal adaption (or PNA) and maturation (or GA). We do so for the 9 features that included both PNA and GA fixed effects, but not the PNA–GA interaction term, as this is not a constant ratio, as Fig. 3(b) shows. Median (IQR) for these ratios, plotted in Fig. 4, are 1.37 (1.30 to 2.04) days/weeks with a range of 1.01 to 2.65 days/weeks. All ratios are positive, meaning the direction of the trend over PNA is in the same direction as the trend for maturation. Thus, the 2.25 days PNA included in our modelling, from 6 to 60 hours PNA, equates to a range of 2.27 to 5.96 weeks of intra-uterine maturation. This relatively large change is also evident in the plots in Fig. 3. For the rEEG lower margin in Fig. 3(a), for example, we can see that at 25 weeks of gestation, the increase after the 2.25 days of postnatal adaption is equivalent to maturation for a 30 week GA infant.

We found no effect for surfactant on the averaged qEEG features (*P* > 0.05, see Supplementary Table S1). Fentanyl–suxamethonium administration also had negligible effect on the qEEG: almost all (33/34) features were not altered by fentanyl, the exception being the *δ*-band EEG skew feature (see Supplementary Table S2). As almost all infants received caffeine (27/28), we could not examine the influence of caffeine on the qEEG in this study. None of the infants in our study received morphine before or during the EEG.

### Example: Algorithm to Estimate EEG Maturational Age

**Figure 5:**
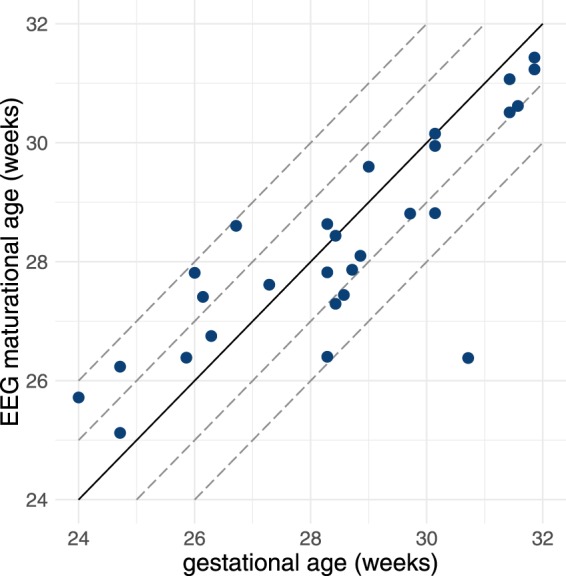
Algorithm’s estimation of EEG maturational age from testing results comparative to gestational age for *n* = 28 infants. Correlation coefficient is *r* = 0.83. Dashed lines represent 1 and 2 weeks deviation from the ideal 1:1 representation (solid line).

The algorithm’s feature set included the 27 features that are dependent on gestational age (see Tables 1 and 3) in addition to PNA as a feature. The SD of the cross-validation testing error between GA and estimated EEG maturation was 9.1 days, compared to an SD of 16.0 days for the distribution of GA; correlation was 0.83 between known and estimated GA; and 96% (64%) of estimates were within ±2 (±1) weeks. Figure 5 plots the relation between known and estimated maturation, showing similar results to another method developed on a similar cohort^17^. The mean rank of relative influence of PNA was 5 (SD 1, range 2–9) from the total of 28 features. The reduction in performance by random permutation of PNA was 1.8% (SD 0.3%, range 1.1–2.9%).

**Table 3:** Estimates (95% confidence intervals) of fixed-effect coefficients for EEG features in the spectral distribution and inter-hemispheric connectivity groups. Coefficients of the mixed-model include intercept, gestational age (GA, age at birth in weeks), postnatal age (PNA, time after birth in days), and the interaction term between PNA and GA (PNA × GA).

Feature type			Coefficients			
group	Feature	FB	Intercept	PNA (days)	GA (weeks)	PNA × GA
spectral distribution	RSP (%)	*δ*	94 (93, 94)			
		*θ*	5.1 (4.5, 5.9)	−0.38 (−0.73, −0.06)		
		*α*	1.1 (−1.2, 3.4)	−1.7 (−3.4, 0)	7.7e-3 (−73.0e-3, 90.4e-3)	0.058 (−0.002, 0.117)
		*β*	−0.69 (−1.32, −0.05)		0.041 (0.018, 0.063)	
	spectral flatness	*δ*	0.27 (0.26, 0.28)			
		*θ*	0.54 (0.39, 0.69)	9.5e-3 (3.8e-3, 15.7e-3)	7.0e-3 (1.6e-3, 12.2e-3)	
		*α*	0.62 (0.56, 0.69)	9.5e-3 (2.9e-3, 16.0-e3)	7.2e-3 (5.0e-3, 9.4e-3)	
		*β*	0.67 (0.66, 0.68)			
	fractal dimension		0.98 (0.82, 1.12)		0.012 (0.007, 0.018)	
	SEF (Hz)		6.2 (0.9, 11.9)	−3.6 (−7.1, −0.1)	−0.098 (−0.297, 0.089)	0.12 (0.01, 0.24)
connectivity	coherence	*δ*	0.45 (0.31, 0.58)		−0.011 (−0.015, −0.006)	
		*θ*	0.42 (0.33, 0.52)		−0.010 (−0.014, −0.007)	
		*α*	0.38 (0.28, 0.48)		−0.010 (−0.013, −0.006)	
		*β*	0.26 (0.18, 0.34)		−0.006 (−0.009, −0.003)	

Frequency bands (FB): 0.5–3 Hz (*δ*), 3–8 Hz (*θ*), 8–15 Hz (*α*), and 15–30 Hz (*β*). RSP: relative spectral power; SEF: spectral edge frequency.

## Discussion

From a comprehensive set of qEEG features, we find rapid maturation for a subset of features over the first 60 hours after birth that is equivalent to between 2.3–6.0 weeks of intra-uterine maturation. Most (79%) qEEG measures are dependent on GA, which is in keeping with results from other qEEG studies^12–17^. What we show, however, is that the majority (56%) of the features change significantly over the first 3 days after birth. This temporal evolution for many features is consistent with increasing intra-uterine maturation, as indicated by the positive PNA-to-GA ratio for all features in Fig. 4.

This remarkable change in qEEG appears to represent an increase in continuity of the EEG, as continuity increases with maturation^9–11^. Also, of the features from the discontinuous group that included PNA as a dependent variable (7/13), almost all (6/7) showed a progression towards decreasing discontinuous activity. For example, decreasing maximum inter-burst interval in Table 1 indicates decreasing discontinuity of the EEG^11^. The only exception was the EEG-skew feature in the *δ*-band which show increasing trends from GAs <28 weeks and then decreasing trends thereafter (see Supplementary Fig. S1). An increase in continuous activity suggests an increase in active, or rapid-eye movement (REM), sleep^31,32^. Yet without polysomnography to define sleep states, we lack certainty on whether these changes are related to the evolution of the sleep-wake cycle over the first days after birth.

The increase in EEG continuity may reflect a recovery from a suppressed EEG during birth. The birth process is a major stressor for the newborn infant and possibly programmes the response of the hypothalamic-pituitary-adrenal axis for life^22^. A certain degree of birth stress is necessary to provide adequate postnatal adaptation for term infants^33–35^, but the same might be disruptive for the cortical pathway development in the immature preterm brain resulting in altered EEG immediately after birth^36^.

Another explanation is that the significant temporal evolution is a response to medication. No infant received either anticonvulsants or morphine before or during the EEG recording. We found no effect for surfactant on the qEEG; for fentanyl–suxamethonium administration only 1 feature, from the set of 34, was significantly altered and therefore would not change the main findings presented here. We were unable to test the effect of caffeine therapy as almost all (27/28) babies received caffeine. Infants received a loading dose of 20 mg/kg (caffeine per kg of birth weight) followed by a maintenance dose of 10 mg/kg per day as required. Caffeine may influence the EEG: some studies have shown an increase in aEEG continuity in response to caffeine^37,38^, although a recent study found no changes in EEG^39^. A prospective study, with long-term continuous EEG monitoring and a control arm of infants not administered caffeine, is needed to determine the role caffeine may play in this.

Increased cerebral perfusion may be the driving mechanism for the evolving qEEG^25,26^. Different studies–using different measurement techniques–have shown an increase in cerebral blood flow volume over the first 2 to 3 days. These techniques include xenon-clearance methods^40^, ultrasound flowmetry^41^, and changes in oxygenated haemoglobin as measured by near infrared spectroscopy (NIRS) in response to changes of the fraction of inspired oxygen for ventilated infants^42,43^. Other studies, using measures such as NIRS-derived cerebral oxygenation^44–47^ and superior vena cava flow^48^, also point to increased cerebral perfusion. For most of these studies the increase in cerebral blood flow (or oxygenation) is most prominent over days 1 and 2^40–43,45,47^, with a smaller increase, or even decrease, on day 3^40,41,45,47^. Although animal studies have shown that the local blood flow is not associated with neural activity in the developing brain^49^, it seems reasonable that the global nature and magnitude of increased blood flow during transition to the extra-uterine environment may induce increased neuronal activity. These changes in cerebral perfusion may represent normal cardiovascular adaption to postnatal life^42,50^.

These findings have implications for future studies: the time-varying nature of the EEG should be integrated into analysis of the EEG during this transitional period. For example, when using qEEG to compare between difference groups, PNA should be uniform between groups. If reporting qEEG values, PNA should also be reported. Similar consideration should also be applied to automated methods. For example in the maturation estimator developed here, we find that PNA is an important feature in the algorithm, ranked on average as the 5th most important feature from the set of 28.

This evolving EEG may also affect the visual interpretation of preterm EEG. For example, our results show that PNA alters the duration of maximum inter-burst intervals to the extent of almost 1 s/day, which is equivalent to 1.23 weeks of maturation. These changes may, in part, explain the large variability in reported maximum inter-burst intervals in the literature^10^. An evolving EEG may also alter grading of the EEG for abnormalities^5,51^. For example, dysmature patterns, defined as ≥2 weeks delayed maturity, are associated with chronic stage abnormalities and used during visual grading of the EEG to better define the prognosis. Many features, over the 2.25 days of recordings, change more than 2 weeks in maturation; see Fig. 4. Thus, it is plausible that the influence of PNA at EEG recording could falsely indicate dysmaturity for a normal EEG.

Although many studies have analysed extra-uterine maturation with serial EEG, for example Niemarkt *et al*. and Shany *et al*.^52,53^, only a few studies have reported changes in a limited qEEG feature set^25–27^; some of these findings overlap with ours. We find a similar direction of evolution for *δ*-power and relative *θ*- and *α*-power in studies by Victor *et al*.^26^ and Schumacher *et al*.^27^, but not in other absolute and relative frequency bands. Similar direction of evolution also for spectral edge frequency in studies by West *et al*.^25^ and Victor *et al*.^26^, although our modelling includes a PNA–GA interaction term (see Supplementary Fig. S1). And similar again for percentage of inter-burst intervals (inverse of our burst ratio measure) and maximum inter-burst interval (90th centile)^26^; and median amplitude (similar to our median rEEG measure)^25^. It is difficult to be more specific in our comparisons for various reasons. First, we selected a cohort without brain injuries and normal neurodevelopment at 2 years of age; other studies did not include neurodevelopmental outcome^25–27^. Second, methods for generating qEEG will differ; for example Victor *et al*.^26^ identified bursts and inter-bursts by visual interpretation on the EEG, whereas we used an automated method^54^ (although the automated method correlates well with the human interpretation). Also, there may be differences in how qEEG measures are defined. For example, West *et al*.^25^ used a medium amplitude measure from the aEEG, but we use rEEG as aEEG is not clearly defined^30^. The advantage of using the NEURAL feature set^30^, as we did here, is that all features are clearly defined with full implementation details, including open-source code^55^ to enable reproducible research and fair comparisons between different studies. Third, our modelling approach allows us to quantify the linear trend of the feature over time, beyond just testing for an increase or decrease as previous studies have done^25–27^. For example, median rEEG increases by 5.2 *μV*/day (95% CI: 3.4 to 7.1 *μV*/day). Including GA in the model, when appropriate, relates this trend in the context of the GA of the infant. For example, the maximum IBI decreases by 0.99 s/day (95% CI: 1.84 to 0.11 s/day) with PNA, but also decreases by 0.81 s/week (95% CI: 1.23 to 0.44 s/week) with GA.

Other factors strengthen our study over previous work. First, although the 1-h epochs were selected as being predominately artefact free, we include an automated method to remove further artefacts, if present^30^. This was identified by Schumacher *et al*.^27^ and Victor *et al*.^26^ as a missing component of their analysis, and a potential source of error when estimating spectral-edge frequency by West *et al*.^25^. Second, we include multiple time points (6 in total) over the first 3 days after birth, whereas previous studies included only 3, 1 per day. Third, we present a comprehensive set of qEEG features, including automated estimation of burst and inter-burst features (for example maximum IBI), measures of inter-hemispheric connectivity (coherence), higher-order moments of the EEG to represent discontinuity (skew and kurtosis), and measures of spectral shape (spectral entropy and fractal dimension); in addition to spectral power (absolute and relative) and voltage peak-to-peak measures (rEEG) as presented elsewhere^25–27^.

Although different sleep states will affect the qEEG, we did not include state annotations in our analysis for 2 reasons. First, the duration of the epoch (1 h) is sufficient to incorporate different states^25,32^. Second, we consider that the sampling of the EEG into epochs to be representative of the continuous recording. We determined sampling times (at 6, 12, 24, 36, 48, and 60 h PNA) before reviewing the EEG and selected epochs as close as possible to these time points, without regard to sleep states. Therefore, we think it unlikely that any sampling error will introduce bias in our results.

A limitation of our study is the moderate numbers of infants (*n* = 28) included in the analysis. The larger the cohort the stronger the inferences drawn; however, generating a large data set with continuous, long-duration EEG recordings from hours after birth with 2-year neurodevelopment assessment in a cohort of preterm infants <32 weeks of gestation requires significant effort and resources. Yet even with the moderate sample size, the mixed-effects models do find statistical significant (*P* < 0.05) linear trends for PNA and GA. Another limitation is that we analyse only the immediate postnatal period, up to 2.5 days. We expect that this rapid period of adaption will taper into the more moderate evolution of extra-uterine maturation^52,53^ or may even return to a baseline level. For this we would need serial EEG recordings to model a longer duration after birth, within the first week for example, including non-linear mixed effect models to account for the expected slowing of this rapid adaption period.

In conclusion, we find rapid evolution in the normal trajectory of qEEG over the first days of life for the preterm infant. The rate of change of the qEEG is significantly higher than intra-uterine changes over the same period. Post-natal adaption of the EEG must be considered when interpreting EEG recordings. The trajectories of EEG within this period can, potentially, be used as an additional biomarker for the identification of infants with disrupted neurological function, often caused by haemodynamic instability during postnatal transition, and complement longitudinal studies of the EEG during the preterm period for the prediction of neurodevelopmental outcome.

## Methods

### Patients

EEG data was retrospectively selected from a cohort of preterm infants recruited between 2009 and 2011 in the NICU of the Cork University Maternity Hospital, Ireland. Preterm infants <32 weeks of gestation had continuous video and EEG monitoring over the first 3 days after birth. Data collection was approved by the Cork Research Ethics Committee of Cork Teaching Hospitals, Ireland. Informed and written parental consent was obtained before EEG monitoring. Data collection and all methods in this study were conducted in accordance with the principals of the International Conference on Harmonisation of Technical Requirements for Registration of Pharmaceuticals for Human Use of Good Clinical Practice (ICH-GCP).

Preterm infants were included in this study if they met the following criteria:

- GA <32 weeks;
- Apgar ≥6 at 5 minutes after birth;
- absence of congenital brain malformations, chromosomal or genetic aberrations, or inborn errors of metabolism;
- absence of grade II or higher intra-ventricular haemorrhage (IVH) and absence of periventricular leukomalacia (PVL);
- absence of major comorbidities (such as sepsis, necrotising enterocolitis, cardiac dysfunction, bronchopulmonary dysplasia) at time of EEG recording;
- no anti-epileptic drugs before or during EEG recording;
- EEG monitoring within 72 h from birth;
- favourable outcome at 2 years of age or adverse outcome ascribable to later morbidities with respect to timing of EEG recording.

Outcome was assessed by the Bayley Scales of Infant Development-III at 2 years corrected age. We define favourable outcome as all three Bayley subscales (motor, cognitive, and language) within one standard deviation (SD) of the standardised mean score. IVH and PVL are assessed by cranial ultrasound as part of routine clinical care within the first few days after birth.

### EEG recording

EEG was recorded using the NicoletOne device (Natus Medical Inc., Pleasanton, CA, USA) with 11 electrodes placed according to the international 10–20 system of electrode configuration. Electrodes covered the frontal, central, temporal, and occipital regions, with a reference electrode at Fz and a ground electrode behind the left ear^56^. Recording started after birth when clinical staff determined the infant was stable and continued for up to 72 h after birth.

### EEG features

One-hour epochs were pruned from the EEG at 6, 12, 24, 36, 48, 60, and 72 h after birth. Epochs that were significantly corrupted by artefact, as determined by a neonatal EEG expert (EP), were eliminated from further analysis. An automated method removed periods of remaining artefacts, if present, in the EEG^30^. We applied these rule-based methods, using default settings, to identify and eliminate (1) improper electrode placement, (2) electrode coupling, (3) impedance testing, (4) high-amplitude artefacts, and (5) impulse-like activity^30^. After artefact removal, the EEG was low-pass filtered to 30 Hz and then down-sampled from 256 Hz to 64 Hz. Quantitative features were then computed on the bipolar montage of F4-C4, C4-O2, F3-C3, C3-O1, T4-C4, C4-Cz, Cz-C3, and C3-T3.

**Figure 6:**
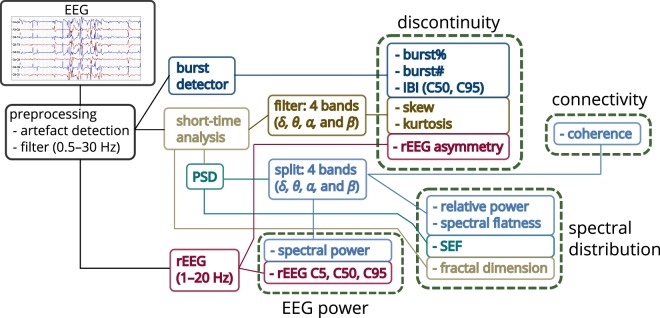
Outline of process to generate quantitative EEG. Features grouped as measures EEG power, spectral distribution, connectivity, and discontinuity. Frequency bands (FB): 0.5–3 Hz (*δ*), 3–8 Hz (*θ*), 8–15 Hz (*α*), and 15–30 Hz (*β*); C5, C50, and C95: 5th, 50th, and 95th centiles; SEF: spectral edge frequency; rEEG: range EEG; IBI: inter-burst interval; burst%: burst ratio; burst#: number of bursts.

Features were computed using the NEURAL toolbox^30^ and grouped into measures of EEG power, discontinuity, spectral distribution, and inter-hemispheric connectivity, as shown in Fig. 6. The power-feature group contains spectral power and range-EEG (rEEG)^14,16^ features. The rEEG is a measure of peak-to-peak voltage developed as an alternative to amplitude-integrated EEG (aEEG)^14,16^. Median rEEG and the lower- and upper-margin (5th and 95th centiles) of the rEEG were used as features in this group. The discontinuity-feature group contains higher-order moments of the EEG signal (skew and kurtosis), asymmetry of the rEEG^14^, and features of the burst annotation^17^. The spectral-feature distribution group contains fractal dimension, spectral-edge frequency, spectral flatness (spectral Wiener entropy), and relative spectral power. And the inter-hemispheric connectivity group includes coherence of channels across hemispheres. Figure 6 outlines some details of the implementation with full details elsewhere^30^.

The power spectral density estimate (PSD), used to generate the frequency-domain features (see Fig. 6), is implemented with an 8 s Hamming window at 75% overlap. Features generated using the PSD, expect for the spectral edge frequency, were estimated within four frequency bands, namely 0.5–3 Hz (*δ*-band), 3–8 Hz (*θ*-band), 8–15 Hz (*α*-band), and 15–30 Hz (*β*-band)^30,54,57^. EEG kurtosis and skewness are also generated for these 4 frequency bands. The rEEG is generated within a 1–20 Hz bandwidth. For both the rEEG, and the EEG skewness and kurtosis features, band-pass filtering is implemented using a 5th-order infinite-impulse response (IIR) filter with a forward–backwards procedure to ensure zero phase.

Connectivity was assessed by quantifying coherence between similar regions on both hemispheres, for example between F4-C4 and F3-C3. Zero coherence was estimated using an analytic expression of the upper 95% CI under the hypothesis of independence^58^. The auto- and cross-PSD were generated for the coherence function using an 8 s rectangular window with no overlap^58^. And lastly, fractal dimension was computed using the Higuchi method with a maximum scale value of 6^59^.

Bursts and inter-bursts features are derived from an annotation of the bursts for each channel of EEG. An automated method generates the burst annotation for the 1-h epochs^54^. Four features are then computed from this annotation: number of bursts, percentage-time of bursts (burst ratio), and median and maximum (95th centile) duration of inter-bursts (inter-burst interval, IBI). The method’s broad definition of a burst includes any EEG waveform not defined as an inter-burst^54^. This differs to the terminology commonly used in electrophysiology^60^ and therefore the method will classify continuous activity as long-duration bursts. Thus, we expect the burst ratio to be greater (and number of bursts to be less) in continuous, compared to discontinuous, activity.

All time-domain and frequency-domain (PSD-based) features are estimated within a 32 s segment with 50% overlap (short-time analysis in Fig. 6). The median is used to summarise the segments over the 1-h epoch, in each channel. rEEG and burst features are estimated on the full 1-h epoch. The median is used again to summaries the features, excluding coherence, over all channels. The total set consists of 34 features, 24 of which are estimated on the 4 frequency bands (*δ*, *θ*, *α*, and *β* bands).

### Modelling temporal evolution

We fit a linear mixed-effects model for each feature to assess the temporal evolution over the first 72 h after birth. Fixed effects for the models include intercept, postnatal age (PNA), GA, and PNA–GA interaction terms. Random effects include intercept and PNA terms. PNA is measured in hours after birth over the first 3 days of life. GA, a measure of maturation, is the duration from the first day of a woman’s last menstrual period to birth. We include GA because many qEEG measures are dependent on maturation^14,15,17,61–64^.

We use a backwards selection procedure for model selection. Starting with the most complex model which includes all fixed and random effects, we systematically remove random and fixed effects if the more complex model is not a significant improvement over the simpler model^4,65^. We use the log-likelihood, with the *χ*^2^-distribution at *P* < 0.05, to assess the potential improvement in model fit. Backward selection starts with the random effects, first random PNA and then intercept. Next, the fixed effects: PNA–GA interaction, then GA, then PNA. If the PNA–GA interaction term is included in the model, both PNA and GA fixed effects are also included. Confidence intervals for the fixed effects are estimated using a bootstrap with 1,000 iterations. Each feature has a different model.

The PNA coefficient captures the immediate evolution over first few days of transition to extra-uterine life as a linear increase or decrease (trend). The GA coefficient quantifies the linear trend for intra-uterine maturation. When both PNA and GA coefficients are included the overall trend is represented as a linear plane in 2D space. And the PNA–GA interaction term quantifies the influence of GA on this 2D plane, warping it to a nonlinear plane.

To determine if medications influence the temporal evolution of the qEEG, we examine the effect of early medications–specifically morphine, fentanyl, caffeine, and surfactant replacement therapy–on the qEEG^66^. Fentanyl, combined with the muscle relaxant suxamethonium, is administered before endotracheal intubation and caffeine is administered in response to apneic events or before extubation. As our data set is small, we do not include these potential confounders in the mixed-effects models to avoid over-fitting the models. Instead we employ multiple linear regression to test the effects of these potential confounders on the qEEG. GA and medication usage, as a binary variable (yes/no), are included as the independent variables with the qEEG feature, averaged over the PNA epochs, as the dependent variable. The medication coefficient is determined significant using the log-likelihood ratio test comparing it to a model without the coefficient. Confidence intervals (95%) are generated for the potential confounder.

### Automated analysis of EEG maturational age

To further test the hypothesis that short-term postnatal changes in cerebral activity is significant in terms of the long-term intra-uterine maturation, we evaluate the importance of PNA in an algorithm that estimates the maturation of the EEG. Automated methods, using qEEG and machine learning, have recently been developed to estimate EEG maturational age^7,17^. These methods are trained and tested on the EEG using GA as a marker of EEG maturational age. Here we develop a new model combining qEEG and PNA as features and determine if PNA is central to the model’s performance.

The method uses the proposed qEEG set, providing the feature is dependent on GA. Dependence is determined from the mixed-effect models described in the previous section. These features, including PNA, are then combined in a regression algorithm to relate to maturation, as measured by GA^7,17^. (PNA was used as a feature in O’Toole *et al*.^17^ but its importance was not assessed.) We use a gradient-boosting algorithm, using the ${\ell }_{1}$-norm loss function with decision trees as the base learner^67^. Parameter values are set following guidelines by Hastie *et al*.^68^: numerous iterations (5,000) and a correspondingly small shrinkage value (0.01) to control over-fitting; tree size of 6 and 1/2 of the data was randomly selected at each iteration. We set these parameters before training and testing the model because in the development stage we found little to no improvement by selecting different parameters. The maturation estimate for each infant is the mean value over the multiple epochs. Training and testing uses a leave-one-out cross-validation scheme and performance reported with the SD of error, Pearson’s correlation coefficient, and percentage of correct classifications of GA within 1 and 2 weeks^17^.

Two measures are used to assess the importance of PNA in the algorithm: relative influence and performance loss caused by random permutation of the feature. Relative influence ranks features based on their contribution to reducing squared error when building the decision trees^67^. Because we set the parameters in the gradient boosting’s algorithm to randomly select one-half of the feature set at each boosting iteration, no 2 models are exactly the same. Thus, we iterate the entire process 1,000 times and estimate the relative importance measures at each iteration.

## Supplementary information

Supplementary Info

## Data Availability

The feature set was generated using Matlab (release R2013b, The MathWorks, Inc., Natick, Massachusetts, United States) with the NEURAL toolbox (version 0.4.0^55^) and the burst detector algorithm (version 0.1.2^69^). Multiple linear regression, the linear mixed-models, and the gradient boosting algorithm were computed using *R* (version 3.3.2, The R Foundation of Statistical Computing, www.r-project.org). The mixed models used the *lme4* (version 1.1.10) package and gradient boosting algorithm used the *gbm* (version 2.1.3) package. The *R* code to generate the mixed-effects models and the maturation estimator, in addition to the qEEG feature set and parameter file used in the NEURAL toolbox, is available at https://github.com/otoolej/postnatal_EEG_evolution (version 1.0.0).
