# Supplementary material for: Temporal evolution of quantitative EEG within 3 days of birth in early preterm infants
Source: Sci Rep. 2019 Mar 19;9:4859. doi: 10.1038/s41598-019-41227-9 (PMC6425040; doi:10.1038/s41598-019-41227-9)
Supplement: Supplementary file 1 — Supplementary Info [file 41598_2019_41227_MOESM1_ESM.pdf]

Temporal evolution of quantitative EEG within 3 days of birth in early preterm infants

John M O' Toole[1,2*], Elena Pavlidis[1], Irina Korotchikova[2], Geraldine B Boylan[1,2], and Nathan J Stevenson[3]

[1]Neonatal Brain Research Group, Irish Centre for Fetal and Neonatal Translational Research (INFANT), University College Cork, Ireland
[2]Department of Paediatrics and Child Health, University College Cork, Ireland
[3]BABA Center, Department of Children's Clinical Neurophysiology, Children's Hospital, HUS Medical Imaging Center, Helsinki University Central Hospital and University of Helsinki, Finland
[*]jotoole@ucc.ie

Temporal evolution with gestational age dependencies

Figure S1 presents a visualisation of the changing temporal trajectories with increasing GA specific to these 6 features. For example, for infants born at 24 weeks of gestation, there is a large increase in δ-band spectral power (approximately 700 μV^2) over the first 60 hours of life. For infants born at 32 weeks of gestation however, there is a small decrease in the spectral power (approximately 50 μV^2) over the same time period.

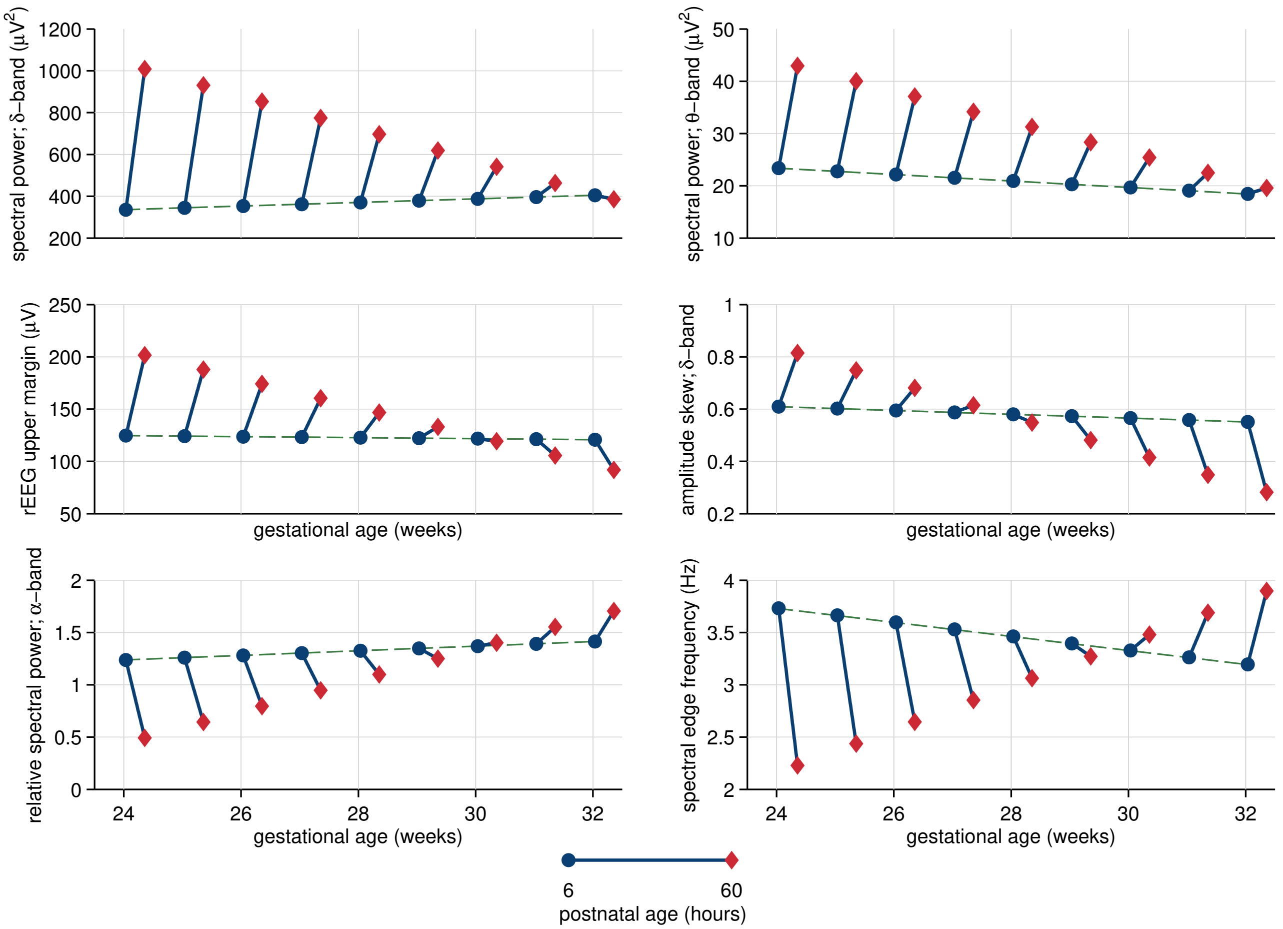

Figure S1. Trajectories of the time fixed-effect (postnatal age, as solid lines) plotted at selected gestational ages. The dashed line represents a baseline at 6 hours PNA.

Medication and qEEG: multiple linear regression

Estimates of the independent variable representing medication therapies: surfactant in Table S1 and fentanyl in Table S2. GA is also included as an independent variable with the time-averaged qEEG feature as the dependent variable.

Table S1. Coefficient estimates for surfactant replacement therapy.

Feature type				Regression coefficient		
group	feature	FB	C_{th}	coefficient	(95% CI)	*P*-value
power	SP (μV^2)	δ		8.2	(-174.5, 193.9)	0.939
		θ		2.6	(-6.9, 11.0)	0.622
		α		0.58	(-1.33, 3.14)	0.624
		β		0.13	(-0.93, 1.1)	0.792
	rEEG (μV)		50th	0.25	(-7.30, 8.34)	0.945
			5th	0.48	(-2.51, 3.07)	0.717
			95th	0.56	(-30.17, 28.07)	0.973
discontinuity	r-AS			0.039	(-0.047, 0.117)	0.251
	skew	δ		0.078	(-0.033, 0.192)	0.134
		θ		0.011	(-0.020, 0.049)	0.438
		α		0.0045	(-0.0007, 0.0119)	0.108
		β		0.018	(-0.017, 0.058)	0.361
	kurtosis	δ		0.45	(-0.75, 1.71)	0.387
		θ		2.8	(-2.5, 8.4)	0.214
		α		2.6	(-1.0, 6.8)	0.176
		β		0.71	(-2.39, 3.85)	0.702
	IBI (s)		95th	-0.45	(-2.57, 1.56)	0.764
			50th	-0.22	(-0.62, 0.27)	0.452
	burst% (%)			0.03	(-8.66, 6.99)	0.995
	burst#			9.2	(-29.0, 72.0)	0.686
spectral distribution	RSP (%)	δ		-0.41	(-2.00, 1.46)	0.650
		θ		0.59	(-0.46, 2.19)	0.367
		α		0.075	(-0.318, 0.39)	0.696
		β		-0.045	(-0.274, 0.101)	0.645
	SF	δ		-0.0077	(-0.0336, 0.0179)	0.569
		θ		0.007	(-0.029, 0.039)	0.671
		α		-0.014	(-0.033, 0.004)	0.073
		β		-0.0051	(-0.0368, 0.0167)	0.698
	SEF (Hz)			0.13	(-0.81, 0.98)	0.764
	FD			0.00064	(-0.03717, 0.03142)	0.972
connectivity	coherence	δ		-0.0010	(-0.0322, 0.0303)	0.949
		θ		0.0070	(-0.0138, 0.0345)	0.559
		α		-0.0025	(-0.0178, 0.0179)	0.792
		β		-0.0091	(-0.0253, 0.0091)	0.279

Frequency bands (FB): 0.5–3 Hz (δ), 3–8 Hz (θ), 8–15 Hz (α), and 15–30 Hz (β); C_{th}: centile; CI: confidence intervals; SP: spectral power; rEEG: range EEG; r-AS: rEEG asymmetry; IBI: inter-burst interval; burst%: burst ratio; burst#: number of bursts. Statistical significance: * for $P < 0.05$.

Table S2. Coefficient estimates of fentanyl (with suxamethonium) therapy.

Feature type				Regression coefficient		
group	feature	FB	C_{th}	coefficient	(95% CI)	*P*-value
power	SP (μV^2)	δ		28	(-187, 244)	0.786
		θ		4.7	(-5.7, 15.0)	0.353
		α		1.3	(-1.0, 3.7)	0.240
		β		0.22	(-0.80, 1.24)	0.655
	rEEG (μV)		50th	-0.11	(-7.37, 7.16)	0.976
			5th	0.16	(-2.53, 2.85)	0.903
			95th	14	(-19, 47)	0.394
discontinuity	r-AS			0.046	(-0.022, 0.114)	0.164
	skew	δ		0.11	(0.01, 0.21)	0.019*
		θ		0.014	(-0.015, 0.043)	0.324
		α		0.00093	(-0.00497, 0.00682)	0.746
		β		0.0025	(-0.0384, 0.0433)	0.901
	kurtosis	δ		0.41	(-0.65, 1.46)	0.427
		θ		3.8	(-0.6, 8.2)	0.075
		α		2.0	(-2.0, 6.0)	0.305
		β		-0.18	(-3.92, 3.56)	0.922
	IBI (s)		95th	-0.89	(-3.92, 2.14)	0.546
			50th	-0.23	(-0.81, 0.35)	0.413
	burst% (%)			0.12	(-9.10, 9.34)	0.978
	burst#			7.2	(-38.7, 53.2)	0.746
spectral distribution	RSP (%)	δ		-0.65	(-2.47, 1.17)	0.464
		θ		0.71	(-0.59, 2.00)	0.263
		α		0.17	(-0.21, 0.55)	0.365
		β		-0.064	(-0.259, 0.131)	0.498
	SF	δ		-0.0052	(-0.0326, 0.0222)	0.698
		θ		0.011	(-0.022, 0.044)	0.502
		α		-0.011	(-0.026, 0.005)	0.154
		β		-0.0041	(-0.0304, 0.0222)	0.749
	SEF (Hz)			0.15	(-0.73, 1.04)	0.722
	FD			0.0032	(-0.0330, 0.0394)	0.857
connectivity	coherence	δ		0.011	(-0.019, 0.041)	0.458
		θ		0.018	(-0.005, 0.041)	0.107
		α		0.0064	(-0.0128, 0.0255)	0.494
		β		0.00035	(-0.01696, 0.01766)	0.967

Frequency bands (FB): 0.5–3 Hz (δ), 3–8 Hz (θ), 8–15 Hz (α), and 15–30 Hz (β); C_{th}: centile; CI: confidence intervals; SP: spectral power; rEEG: range EEG; r-AS: rEEG asymmetry; IBI: inter-burst interval; burst%: burst ratio; burst#: number of bursts. Statistical significance: * for $P < 0.05$.
